# Specific characteristics of the medical history of swallowing before and after application of botulinum toxin in patients with cervical dystonia

**DOI:** 10.6061/clinics/2019/e776

**Published:** 2019-03-25

**Authors:** Tatiana Fonseca Del Debbio Vilanova, Vanderci Borges, Henrique Ballalai Ferraz

**Affiliations:** Setor de Transtornos do Movimento, Departamento de Neurologia, Escola Paulista de Medicina, Universidade Federal de Sao Paulo (UNIFESP), Sao Paulo, SP, BR

**Keywords:** Dysphagia, Dystonia, Voice Symptoms, Botulinum Toxin, Swallowing, Aspiration

## Abstract

**OBJECTIVES:**

To compare signs and symptoms of dysphagia in individuals with cervical dystonia (CD) before and after application of botulinum toxin (BTX).

**METHODS:**

A prospective study was conducted with 20 patients diagnosed with CD with indications for BTX application. We selected 18 patients who met the study inclusion criteria. All individuals were patients from the Movement Disorders Unit, Department of Neurology, Federal University of São Paulo. BTX was applied in the cervical region at the necessary dose for each individual. To identify signs/complaints of changes in swallowing, we used a specific questionnaire that was completed by patients and/or their companions on the day of BTX injection and repeated 10 to 15 days after BTX injection.

**RESULTS:**

Among the 18 study subjects, 15 (83.3%) showed primary and three (16.7%) showed secondary cervical dystonia. The most frequent dystonic movements were rotation (18), tilt (5), forward shift (3), backward shift (7), shoulder elevation (12), shoulder depression (2), and cervical tremor (6). The main complaints reported before BTX application were voice changes in 10 (55.6%), need for adjustment of eating position in 10 (55.6%), coughing and/or choking while eating in nine (50%), and increased eating time in nine (50%) individuals. The main complaints reported after BTX application were coughing and/or choking while eating in 11 (61.1%), voice changes in nine (50%), sensation of food stuck in the throat in eight (44%), and increased eating time in eight (44%) individuals.

**CONCLUSION:**

The administration of a swallowing-specific questionnaire to individuals with CD before and after BTX application enabled the identification of possible dysphagia symptoms prior to drug treatment resulting from CD, which are often subsequently interpreted as side effects of the drug treatment. Thus, dysphagia can be managed, and aspiration symptoms can be prevented.

## INTRODUCTION

Dystonia is defined as “persistent or intermittent muscle contractions, usually involving agonist and antagonist muscles simultaneously, causing abnormal, often repetitive, movements, postures, or both” 1,2. Cervical dystonia (CD) is a focal dystonia that is usually primary and characterized by persistent involuntary contractions of cervical muscles, causing abnormal head movements or postures 3,4. CD is one of the most common forms of dystonia and may cause considerable disability and adversely affect quality of life 5.

Botulinum toxin (BTX) type A is a potent biological neurotoxin that produces transient muscle weakness by irreversible presynaptic inhibition of acetylcholine release. Its clinical use is considered the best treatment for dystonia, and it produces satisfactory results in most cases, albeit with side effects, including dysphagia, dry mouth and throat, and voice changes such as hoarseness, which are frequently described and may limit the treatment of these patients 6,7.

Dysphagia is described as one of the most frequent side effects of BTX injection, with an occurrence rate ranging from 10-90% 5,8,9, which can be reduced using ultrasound and electromyography to guide the BTX injection 8. Despite the large number of citations and high variability of results, few studies have assessed swallowing in these individuals before and after treatment 10-12. Many studies include the description of symptoms of dysphagia as a single item in the general questionnaire of side effects after BTX injection 6,13-15.

Changes in swallowing, such as delayed swallowing reflex and presence of food stasis in the epiglottic vallecula, have previously been described as changes in the pharyngeal phase of swallowing in this population before any therapeutic intervention 16-18. These changes highlight the effects of cervical dystonic movements and their muscle conditions on performing one of the most important functions: eating.

In phase 1 of this study, we aimed to investigate the swallowing perception and complaints of individuals with CD before and after BTX injection in treatment-naive patients to analyze the actual relationships among the disease, swallowing and medication before and after treatment.

## MATERIALS AND METHODS

### Study Participants

This is a case-control study of patients with CD. Twenty patients were randomly selected, according to the flow of patients in the Movement Disorders Unit, Universidade Federal de São Paulo – UNIFESP, São Paulo, Brazil. Two individuals withdrew from the study. Therefore, 18 patients diagnosed with dystonia, with ages ranging from 15 to 45 years and with a median age of 45 years, were evaluated. This study was approved by the Ethics Committee of UNIFESP (process number 0700/03) and by the Research Ethics Committee of the Institute of Education and Research (Instituto de Ensino e Pesquisa – IEP), Sírio Libanês Hospital, São Paulo (process 2005/19). The inclusion criteria were as follows. 1. patients with a confirmed diagnosis of primary or secondary CD based on clinical examination and on neuroimaging, such as computed tomography and/or magnetic resonance imaging; 2. therapeutic indication of BTX application in the craniofacial segment in treatment-naïve patients; 3. agreement to participate in the study after receiving information on the project and signing the informed consent form approved and revised by the Research Ethics Committees of UNIFESP-EPM and of Sírio Libanês Hospital, where the swallowing videofluoroscopy examinations were performed. Patients with concomitant neurological impairments, such as Parkinson's disease, Parkinson's syndrome, cerebral palsy, stroke before onset of dystonia, or ataxias, individuals younger than 13 years, and patients who refused to participate in the study and/or who had previously received BTX were excluded from the study.

### Measures

Neurological evaluation and BTX A injection: All patients were subjected to a neurological medical evaluation for diagnosis, classification, and therapeutic indication of BTX. They were also evaluated and classified before and after the BTX injection using the scale developed by Tsui et al. 19. The doses applied were determined during the medical evaluation. The medication used was abobotulinum toxin (Dysport - Ipsen Ltd., Slough, Berkshire, UK), diluted in 1.25 ml saline. The doses ranged from 360 to 720 U, with a mean of 529.7 total U. Two to three injection points were used per muscle to maximize the effect of the medication.

Speech therapy and evaluation of swallowing: All individuals answered a medical history questionnaire before (two to six days, mean of four days) and after (10 to 15 days, mean of 12 days) BTX injection because the latter period corresponds to the peak treatment effect. The questionnaire included specific and direct questions aimed at identifying swallowing and/or speech complaints. The swallowing-specific medical history questionnaire consisted of 13 closed-ended questions that were answered with “yes” or “no”.

1. Voice complaints: individuals were asked about voice changes following the onset of cervical dystonia. Individuals who answered “yes” were asked to describe the changes they noticed.

2. Voice changes while eating: individuals were asked about voice changes while eating, as an indication of the presence of food in the larynx, which is a symptom of dysphasia.

3. Coughing or gagging while eating: individuals were asked about the presence and frequency of coughing and/or gagging during meals.

4. Difficulties swallowing saliva: individuals were asked about the presence of drooling and difficulties managing or swallowing saliva.

5. Chewing difficulties: individuals were asked about the presence of difficulty and/or changes in mastication after the onset of dystonia.

6. Additional effort required for swallowing solids: individuals were asked about the need for extra effort to swallow solids. If they answered “yes”, this would indicate difficulties with food bolus propulsion, which is one of the possible changes in the oral phase of swallowing.

7. Sensation of food stuck in the throat: individuals were asked about the sensation of food stuck in the throat during or after eating. If they answered “yes”, this would indicate likely inefficient food bolus propulsion, leading to the accumulation of food (stasis) in the oro- and/or hypopharynx, which are possible symptoms of changes in the oral and/or pharyngeal phases of swallowing.

8. Eating fatigue: individuals were asked about worsened fatigue while eating compared to the conditions before disease onset. If they answered “yes”, this would indicate likely difficulties with food bolus propulsion and transport during the oral and pharyngeal phases of swallowing.

9. Increased eating time: individuals were asked about increases in total eating time. If they answered “yes”, this would indicate increased swallowing difficulties and effort.

10. Reduced appetite: individuals were asked about a reduced amount of food ingested associated with a lack of appetite. If they answered “yes”, the complaint of lack of appetite would indicate swallowing difficulties, which are often masked.

11. Complaint of retching: individuals were asked about episodes of regurgitation, heartburn, or acid reflux after eating. If they answered “yes”, this would indicate symptoms of esophageal dysphagia, possibly associated with laryngeal penetration and/or aspiration events after swallowing.

12. Need to adjust posture while eating: individuals were asked about the need for postural changes or for performing a sensory maneuver to momentarily eliminate the dystonic movement to facilitate eating. If they answered “yes”, this would indicate a possible direct association between the dystonic movement and dysphagia, and possible improvement after BTX injection would result in elimination of the movement.

13. Need to adjust eating utensils: individuals were asked about the need to change their usual eating utensils (for example, if they began using a spoon instead of a fork). If they answered “yes”, this would indicate signs of altered oral control of maintaining the food in the mouth and/or altered food transport from the plate to the mouth, thereby contributing to the individuals' lack of interest in eating.

### Data Analysis

The swallowing-specific medical history findings before and after BTX injection were statistically compared using McNemar's test to assess the significance of changes after administrating the medication.

## RESULTS

Eighteen individuals with CD were evaluated, including 16 patients with idiopathic and two with secondary CD. The group consisted of eight men and 10 women, with a mean age of 45 years. The mean age at disease onset was 39 years, and the mean disease duration was 6.5 years.

Primary dystonia was diagnosed in 83.3% (N=15) of cases, whereas secondary dystonia was identified in only three individuals (16.3%). Dystonic rotational movement was identified in all study subjects. Clinical characteristics identified using the Tsui scale were used to categorize individuals as follows: pure rotational torticollis (22.2%, N=4); mixed rotational torticollis (77.7%, N=14), including rotational torticollis and retrocollis (38.8%, N=7), rotational torticollis and anterocollis (16.6%, N=3), and rotational torticollis and laterocollis (22%, N=4); retrocollis (N=0); and dystonic tremor (N=6).

All study subjects showed improved dystonia symptoms post-treatment. The mean dystonia scores according to the Tsui Scale were 9.05 before and 4.72 after BTX injection. Abobotulinum toxin (Dysport - Ipsen Ltd., Slough, Berkshire, UK) was administered with a mean injection dose of 529.77 U, ranging from 360 U to 720 U.

Table 1 shows that significant differences were found for swallowing-specific complaints between the period before and after BTX injection in any questionnaire item, although slight changes were observed in the overall profile of this population.

**Figure 1:**
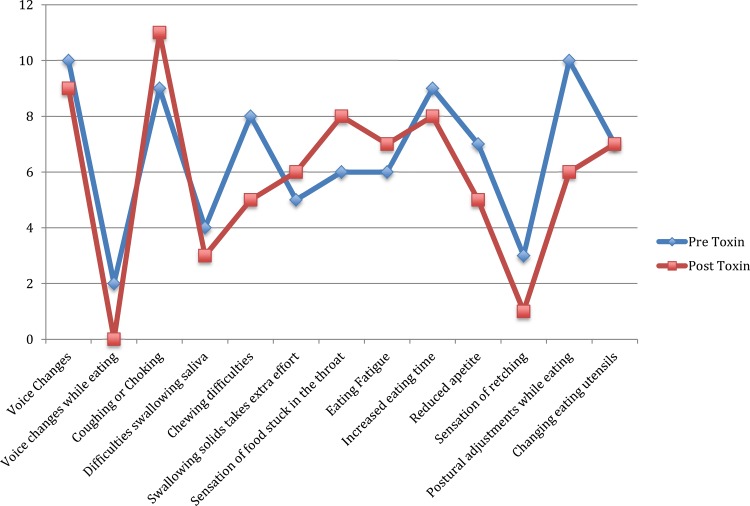
Comparison of swallowing complaints before and after botulinum toxin injection.

Figure 1 shows that the presence of coughing or gagging while eating was the most frequent complaint before 9 and after 11 BTX injection. The curves of both graphs have similar shapes, showing an increase in the number of complaints of requiring extra effort to swallow solids, sensation of food stuck in the throat, and eating fatigue.

## DISCUSSION

Medical history data are usually disregarded in objective studies. In our study, swallowing-specific medical history data collected before and after BTX injection showed that CD patients, when formally and directly questioned, identified changes and reported complaints that often were characteristic of their daily lives. Furthermore, regardless of the degree to which those changes compromised the quality of life of the patients, they became part of their daily routine and often resulted in variable adjustments. Such swallowing and/or voice changes are often not considered to be directly related to dystonia by patients and/or their relatives.

Figure 1 shows the most frequent complaints before BTX injection, including voice changes (55.6%), coughing or choking while eating (50%), increased eating time (50%), need to adjust posture while eating (55.6%) and difficulties chewing (44%).

Phono-articulatory changes in dystonia patients are generally classified as hyperkinetic dysarthrophonia 18,20-22. In our study, complaints of voice changes were observed in 55.6% of study subjects. We suggest that dystonic movements, which may involuntarily occur symmetrically or asymmetrically, cause vocal tract instability, directly affecting parameters such as resonance and voice quality. Furthermore, interruption of the continuous airflow for sustained vocalization can also promote constant breaks in sonority, thus affecting the intelligibility and quality of communication. However, variations in pitch or loudness, inappropriate pauses, hyper or hyponasality, articulatory inaccuracy, and reduced speech rate are characteristic of hyperkinetic dysarthrophonia 18,22-25. Although dysarthrophonia is apparently unrelated to swallowing changes, they maintain a close relationship because the same muscles are involved in these two vital functions. Therefore, if physiological phono-articulatory changes occur, physiological swallowing changes may also occur.

Swallowing complaints, such as choking, when asked about in isolation, are often overlooked because patients only consider choking events when they are sufficiently intense to compromise breathing. Conversely, when combined with coughing episodes while eating, choking may indicate the occurrence of tracheal aspiration, with or without spontaneous exit of the material. A rare study on the preintervention swallowing patterns of patients with CD found that 36% of patients complained of dysphagia 11. In our study, the complaint of presence of coughing and/or choking while eating was reported by nine individuals (50%) before BTX injection and illustrated the presence of eating difficulties before drug treatment in this population. When analyzing the hyperextension posture of individuals or even continuous and involuntary dystonic movements, we must consider that the airway remains open or opens and closes abruptly during swallowing. Therefore, when apnea is necessary, which involves closure of the airway for its protection and synchrony for food bolus transport to the stomach, dystonic movements can interrupt this process and affect postural stability, aggravating the lack of control over food bolus transport and leading to possible food bolus penetration and/or pulmonary aspiration before, during, or after swallowing. However, we believe that the pathophysiology of CD may directly affect swallowing and eating.

The need for postural adjustments while eating was one of the most frequent complaints (55.6%). Many patients reported a fear of hurting themselves with their utensils while eating since the presence of dystonic movements may compromise coordination when transporting food from the plate to the mouth. The same problem was observed by Benecke et al. in 2003 25.

Complaints of chewing difficulties (44.4%) and increased eating time (50%) directly showed the effect of dystonia on the muscles involved in the eating process. Thus, the impact of this disability on the daily life and diet of this population, including food refusal, lack of pleasure during meals, and reduced quality of life, can be analyzed, as it compromises social activities, aggravating reclusion.

Notably, before the drug treatment, a mean of 50% of patients, when directly asked, reported signs of changes or difficulties while eating, although this was not a frequent spontaneous complaint. Few swallowing studies have been conducted before and after BTX injection, and studies reporting swallowing-specific medical history data are even rarer. Comella et al. reported a frequency of 11%, Munchau et al. reported 58.3%, and Dressler et al. reported that 9% of the study population complained of dysphagia before the injection; however, dysphagia complaints were not specified in any of these studies 10,26-27.

After the BTX injection, the mean number of eating-related complaints remained at approximately 50%. The individuals reported the same complaints as those described before the injection. An additional complaint was the sensation of food stuck in the throat, which may reflect a mild worsening of the oral phase of swallowing resulting from changes in food bolus propulsion or in its passage through the oro- and hypopharynx. These changes often derive from changes in pharyngeal propulsion, larynx elevation, and/or opening of the pharyngoesophageal segment. The posterior wall of the pharynx is described as the region most susceptible to BTX migration after its injection into the sternocleidomastoid and scalene muscles 8 and may also contribute to the sensation of food stuck in the throat 8,16,17,28, which is associated with vallecular stasis. A previous study on various types of dystonia reported the occurrence of increased sternocleidomastoid muscle contraction time, increased time of initial elevation and lowering of the larynx, delayed triggering of the swallowing reflex, and increased cricopharyngeal muscle contraction in CD patients, which may in turn compromise and/or affect the dynamics of the pharyngeal phase of swallowing 11.

Coughing and/or gagging complaints increased from 50% pre-BTX to 61.1% post-BTX due to an additional two individuals with this complaint, who also complained of increased sensation of food stuck in the throat, which increased from 33.3% pre-BTX to 44.4% post-BTX. We believe that in both cases, the effect of BTX on the pharynx may explain such complaints, in addition to the heightened attention of these individuals during eating because they were participating in a study on their specific swallowing complaints (Table 1).

However, we observed and concluded that CD alone can affect the swallowing and speaking functions because it is a neurological alteration and compromises both muscle tone and functional aspects of the cervical muscles. The speed, accuracy, and performance of the vital function of eating are compromised. Despite the large functional gain and improved quality of life of these individuals, treatment with BTX may exacerbate pre-existing changes in the phono-articulatory system responsible for speaking and swallowing.

Based on the possible pretreatment alterations, we suggest that CD patients could be subjected to a swallowing evaluation before the drug intervention, thereby enabling the medical team to advise them on postural adjustments and safer food consistencies during peak BTX activity. Thus, alertness, knowledge, and food safety measures can prevent complications resulting from possible tracheal aspirations and can promote safe and functional food intake while maintaining hydration and nutritional support.

We suggest that future studies compare the specific swallowing complaints in individuals with CD pre- and post-treatment with BTX and perform an objective swallowing study of these individuals.

## AUTHOR CONTRIBUTIONS

Vilanova TFDD is the main author, was responsible for the application of the swallowing inventory questionnaire, interpretation of data and manuscript writing. Ferraz HB, is the advisor and reviewer of the research part of the doctorate, and was involved in the manuscript writing. Borges V was responsible for the medical evaluation before and after botulinum toxin application, and manuscript writing.

## Figures and Tables

**Table 1:** Comparison of patient clinical voice and swallowing complaints before and after botulinum toxin injection (N=18).

	Pre-toxin		Post-toxin	
	N	%	N	%
Voice				
change in voice quality	10	56%	9	50%
change in voice quality during eating	2	11%	0	0%
Swallowing				
coughing and/or choking during eating	9	50%	11	61%
difficulty swallowing saliva	4	22%	3	17%
difficulty chewing	8	44%	5	28%
need for hard swallowing	5	28%	6	33%
residual sensation in the throat	6	33%	8	44%
tired sensation after eating	6	33%	7	39%
requiring a long time to eat	9	50%	8	44%
reduced appetite	7	39%	5	28%
frequent regurgitation	3	17%	1	6%
need for an adapted position during eating	10	56%	6	33%
need for adaptation of cutlery during eating	7	39%	7	39%

## References

[1] Fahn S (1988). Concept and classification of dystonia. Adv Neurol.

[2] Dystonia Medical Research 2018 available at https://www.dystonia-foundation.org

[3] Vinken PJ, Bruyn GW (1968). Handbook of clinical neurology.

[4] Andrade LAF, Ferraz HB (1992). Idiopathic dystonia. Clinical profile of 76 Brazilian patients. Arq Neuropsiquiatr.

[5] Contarino MF, Van Den Dool J, Balash Y, Bhatia K, Giladi N, Koelman JH (2017). Clinical Practice: Evidence-Based Recommendations for the Treatment of Cervical Dystonia with Botulinum Toxin. Front Neurol.

[6] Bledsoe IO, Comella CL (2016). Botulinum Toxin Treatment of Cervical Dystonia. Semin Neurol.

[7] Fernandez HH, Pappert EJ, Comella CL, Evidente VG, Truong DD, Verma A (2013). Efficacy and Safety of IncobotulinumtoxinA in Subjects Previously Treated with Botulinum Toxin Versus Toxin-Naïve Subjects with Cervical Dystonia.. Tremor Other Hyperkinet Mov.

[8] Hong JS, Sathe GG, Niyonkuru C, Munin MC (2012). Elimination of dysphagia using ultrasound guidance for botulinum toxin injections In cervical dystonia. Muscle Nerve.

[9] Shahidi G, Mir AP, Shahidi RK, Balmeh P (2013). Severe Dysphagia After Inferior Alveolar Nerve Block Preceded By Cervical Botolinum Toxin Injection: A Case Report. Iran Red Crescent Med J.

[10] Dressler D, Paus S, Seitzinger A, Gebhardt B, Kupsch A (2013). Long-term efficacy and safety of incobotulinumtoxinA injections in patients with cervical dystonia. J Neurol Neurosurg Psychiatry.

[11] Ertekin C, Aydogdu I, Seçil Y, Kiylioglu N, Tarlaci S, Ozdemirkiran T (2002). Oropharyngeal swallowing in craniocervical dystonia. J Neurol Neurosurg Psychiatry.

[12] Ertekin C, Aydogdu I, Ozdemirkiran T, Seçil Y, Bor S (2005). Preswallowing dystonia. Dysphagia.

[13] Jankovic J, Adler CH, Charles D, Comella C, Stacy M, Schwartz M (2015). Primary results from the cervical dystonia patient registry for observation of onabotulinumtoxinA efficay (CD PROBE). J Neurol Sci.

[14] Patterson A, Almeida L, Hess CW, Martinez-Ramirez D, Okun MS, Rodriguez RL (2016). Occurrence of Dysphagia Following Botulinum Toxin Injection in Parkinsonism-related Cervical Dystonia: A Retrospective Study. Tremor Other Hyperkinet Mov.

[15] Poewe W, Burbaud P, Castelnovo G, Jost WH, Ceballos-Baumann AO, Banach M (2016). Efficacy and safety of abobotulinumtoxinA liquid formulation in cervical dystonia: A randomized-controlled trial. Mov Disord.

[16] Zesiewicz TA, Stamey W, Sullivan KL, Hauser RA (2004). Botulinum toxin A for the treatment of cervical dystonia. Expert Opin Pharmacother.

[17] Slawek J, Madalinski MH, Maciag-Tymecka I, Duzynski W (2005). [Frequency of side effects after botulinum toxin A injections in neurology, rehabilitation and gastroenterology]. Pol Merkul Lekarski.

[18] Kreisler A, Verpraet AC, Veit S, Pennel-Ployart O, Béhal H, Duhamel A (2016). Clinical Characteristics of Voice, Speech, and Swallowing Disorders in Oromandibular Dystonia. J Speech Lang Hear Res.

[19] Tsui JK, Eisen A, Mak E, Carruthers J, Scott A, Calne DB (1985). A pilot study on the use of botulinum toxin in spasmodic torticollis. Can J Neurol Sci.

[20] Cannito MP, Doiuchi M, Murry T, Woodson GE (2012). Perceptual structure of adductor spasmodic dysphonia and its acoustic correlates. J Voice.

[21] Rojas GVE, Ricz H, Tumas V, Rodrigues GR, Toscano P, Aguiar-Ricz L (2017). Vocal Parameters and Self-Perception in Individuals With Adductor Spasmodic Dysphonia. J Voice.

[22] Barkmeier-Kraemer JM, Clark HM (2017). Speech-Language Pathology Evaluation and Management of Hyperkinetic Disorders Affecting Speech and Swallowing Function. Tremor Other Hyperkinetic Mov.

[23] Langeveld TP, van Rossum M, Houtman EH, Zwinderman AH, Briaire JJ, Baatenburg de Jong R (2001). Evaluation of voice quality in adductor spasmodic dysphonia before and after botulinum toxin treatment. Ann Otol Rhinol Laryngol.

[24] Boutsen F, Cannito MP, Taylor M, Bender B (2002). Botox treatment in adductor spasmodic dysphonia: a meta-analysis.

[25] Bernecke R, Moore P, Dressler D, Naumann M, Moore P, Naumann M (2003). Cervical and axial dystonia. Handbook of Botulinum toxin treatment.

[26] Comella CL, Tanner CM, DeFoor-Hill L, Smith C (1992). Dysphagia after botulinum toxin injections for spasmodic torticollis: clinical and radiologic findings. Neurology.

[27] Münchau A, Good CD, McGowan S, Quinn NP, Palmer JD, Bhatia KP (2001). Prospective study of swallowing function in patients with cervical dystonia undergoing selective peripheral denervation. J Neurol Neurosurg Psychiatry.

[28] Borodic GE, Joseph M, Fay L, Cozzolino D, Ferrante RJ (1990). Botulinum A toxin for the treatment of spasmodic torticollis: dysphagia and regional toxin spread. Head Neck.

